# An Integrated Web-Based Mental Health Intervention of Assessment-Referral-Care to Reduce Stress, Anxiety, and Depression in Hospitalized Pregnant Women With Medically High-Risk Pregnancies: A Feasibility Study Protocol of Hospital-Based Implementation

**DOI:** 10.2196/resprot.4037

**Published:** 2015-01-16

**Authors:** Dawn Kingston, Selikke Janes-Kelley, Janie Tyrrell, Lorna Clark, Deena Hamza, Penny Holmes, Cheryl Parkes, Nomagugu Moyo, Sheila McDonald, Marie-Paule Austin

**Affiliations:** ^1^University of AlbertaEdmonton, ABCanada; ^2^Alberta Health ServicesEdmonton, ABCanada; ^3^Alberta Health ServicesCalgary, ABCanada; ^4^University of New South WalesSydneyAustralia; ^5^St. John of God HealthcareSydneyAustralia

**Keywords:** Web-based, screening, cognitive behavior therapy, pregnancy, depression, anxiety, psychological stress, quasi-experimental studies

## Abstract

**Background:**

At prevalence rates of up to 40%, rates of depression and anxiety among women with medically complex pregnancies are 3 times greater than those in community-based samples of pregnant women. However, mental health care is not a component of routine hospital-based antenatal care for medically high-risk pregnant women.

**Objective:**

The purpose of this study is to evaluate the effectiveness and feasibility of the hospital-based implementation of a Web-based integrated mental health intervention comprising psychosocial assessment, referral, and cognitive behavioral therapy (CBT) for antenatal inpatients.

**Methods:**

This study is a quasi-experimental design. Pregnant women are eligible to participate if they are (1) <37 weeks gestation, (2) admitted to the antenatal inpatient unit for >72 hours, (3) able to speak and read English or be willing to use a translation service to assist with completion of the questionnaires and intervention, (4) able to complete follow-up email questionnaires, (5) >16 years of age, and (6) not actively suicidal. Women admitted to the unit for induction (eg, <72-hour length of stay) are excluded. A minimum sample of 54 women will be recruited from the antenatal high-risk unit of a large, urban tertiary care hospital. All women will complete a Web-based psychosocial assessment and 6 Web-based CBT modules. Results of the psychosocial assessment will be used by a Web-based clinical decision support system to generate a clinical risk score and clinician prompts to provide recommendations for the best treatment and referral options. The primary outcome is self-reported prenatal depression, anxiety, and stress symptoms at 6-8 weeks postrecruitment. Secondary outcomes are postpartum depression, anxiety, and stress symptoms; self-efficacy; mastery; self-esteem; sleep; relationship quality; coping; resilience; Apgar score; gestational age; birth weight; maternal-infant attachment; infant behavior and development; parenting stress/competence at 3-months postpartum; and intervention cost-effectiveness, efficiency, feasibility, and acceptability. All women will complete email questionnaires at 6-8 weeks postrecruitment and 3-months postpartum. Qualitative interviews with 10-15 health care providers and 15-30 women will provide data on feasibility and acceptability of the intervention.

**Results:**

The study was funded in September, 2014 and ethics was approved in November, 2014. Subject recruitment will begin January, 2015 and results are expected in December, 2015. Results of this study will determine (1) the effectiveness of an integrated Web-based prenatal mental health intervention on maternal and infant outcomes and (2) the feasibility of implementation of the intervention on a high-risk antenatal unit.

**Conclusions:**

This study will provide evidence and guidance regarding the implementation of a Web-based mental health program into routine hospital-based care for women with medically high-risk pregnancies.

## Introduction

### Background

Depression, anxiety, and stress are among the most common morbidities in pregnancy [1-3]; at prevalences of 14% to 25%, they rival the rates of prenatal medical complications such as gestational diabetes [4] and hypertension [5]. Without early intervention, up to 70% of those with prenatal depression or anxiety [6] experience chronic symptoms that extend through the postnatal [2,7,8] and early childhood periods [9-11]. Indeed, recent systematic reviews of pregnancy cohort studies examining early life determinants of adverse child outcomes suggest that prenatal mental illness is one of the main predictors of suboptimal child mental health and development [12,13].

Few studies have explored mental health rates and needs in women hospitalized with high-risk pregnancies. Available research suggests that these women represent a vulnerable group with rates of anxiety and depression up to 40%—more than 3 times greater than those reported in community-based samples of pregnant women [14-16]. Despite high prevalence of symptoms of anxiety and depression, a recent study reported low treatment rates (5%) among high-risk pregnant women despite their inpatient status [15]. In this same study, 77% of women expressed the desire for weekly in-hospital group psychotherapy [15], highlighting the need for regular mental health support. Thus, there is a need to address the mental health needs of hospitalized pregnant women with a low-resource, sustainable approach that can be embedded into routine hospital care.

### Major Impediments to the Delivery of Prenatal Mental Health Care

Barriers to the delivery of prenatal mental health care are ubiquitous across community- and hospital-based settings. In the absence of routine, standardized screening as a component of prenatal care, prenatal mental illness is underdetected and undertreated. Less than one-third of women with depression and anxiety are detected by obstetrical providers [17] and fewer than 20% of women screened as positive follow-up on a referral [18] or engage in treatment [19]. Thus, although there is general consensus about the value of mental health care among prenatal care providers [20-23] and this is supported by international and professional organizations [24-26], serious system-related barriers deter the incorporation of mental health screening, referral, and treatment into the practice of routine prenatal care. Providers cite lack of time, skills (including screening tool selection and use), and established referral systems as the most prominent barriers [27]. To add to this challenge, although pregnant women report high acceptability of provider-initiated mental health screening [28-30], the vast majority express discomfort with self-initiating discussions related to mental health concerns with their health care provider due to stigma, not wanting to take antidepressants, and not understanding whether their symptoms are outside the range of “normal” within the context of pregnancy [31-33].

### Evidence-Based Strategies for Improving Perinatal Mental Health Care

#### Overview

A growing body of evidence based on depression care in the general population suggests that 2 key strategies for reducing barriers to mental health care are (1) employing models of integrated mental health care and (2) Web-based delivery of mental health care. Both of these strategies have high utility for the perinatal period.

#### Integrated Perinatal Mental Health Care

Mental health care is a 3-stage process involving screening, referral, and treatment. Barriers encountered at any of the 3 stages can impede women from achieving treatment success [18]. An integrated approach that seamlessly links mental health screening results to a defined referral process and treatment is a more clinically and cost-effective means for managing depression and anxiety by optimizing treatment accessibility, completion, and response [15,34-36]. Among the few studies that have evaluated the effectiveness and feasibility of models of postnatal depression care with some level of integration [37-42], integrated care results in increased screening and treatment rates with improved clinical outcomes (eg, reduction of postpartum depression). However, only 1 study to date is evaluating an integrated model of prenatal screening, referral, and treatment (trial in progress) [43].

#### Web-Based Delivery of Mental Health Care

##### Web-Based Psychosocial Assessment

Web-based psychosocial assessment can address barriers related to limited time; thus, it is a feasible option for high-paced clinical settings. It offers a standardized approach to assessment, can be adapted for use in populations with low literacy through the addition of audio or video components, can be linked with electronic medical record systems [35,44,45], and is preferred by some patients because it offers an anonymity that an in-person assessment cannot achieve. [45-47]. Pregnant and postnatal women report that Web-based screening is acceptable for sensitive issues, including intimate partner violence [48,49] and mental health [45,50]. Because mental health assessment alone cannot directly improve symptoms [51] or promote treatment engagement [43,52], it must be linked to a defined referral system.

##### Web-Based Clinical Decision Support Systems

Web-based clinical decision support systems promote evidence-based, personalized care by generating ideal treatment and referral options based on a patient’s risk profile [35]. They can be highly beneficial in prenatal mental health care because many perinatal care providers cite lack of knowledge as a barrier to treating mental health problems directly and lack of established linkages with psychological or psychiatric services as a barrier to referral [18].

##### Web-Based Cognitive Behavioral Therapy

Group-based cognitive behavioral therapy (CBT) is an effective intervention for reducing postpartum depression [53-57], but its accessibility is limited by expense and prolonged wait times that extend beyond the prenatal period [58]. Web-based CBT is clinically and cost-effective [59-62], accessible [59], and recommended as a primary therapy for mild and moderate depression [24,63]. Given that a major concern with psychological therapies is nonadherence, a benefit of Web-based CBT is its lower attrition rates (20%) compared to group-based CBT (40%-50%) [10,34,64]. Early evidence suggests that Web-based CBT is effective for reducing postnatal depression [34]. However, to our knowledge only 1 in-progress trial is evaluating Web-based CBT during pregnancy [43]. With clear potential benefits due to low cost, high accessibility, and greater treatment adherence [34], there is a need to determine the effectiveness and acceptability of Web-based CBT in high-risk antenatal inpatients.

### Purpose of the Study and Research Questions

This study is an extension of an in-progress community-based randomized controlled trial (RCT), the Integrated Maternal Psychosocial Assessment to Care Trial (IMPACT) [43], which is evaluating the clinical- and cost-effectiveness of a Web-based mental health care intervention in primary care settings. Initiated by the recruiting hospital, the current study evaluates both the effectiveness of the Web-based mental health intervention in high-risk antenatal inpatients and the feasibility of its integration into the hospital setting. As such, this study is distinguished from the IMPACT trial in its focus on determining the intervention effectiveness in an underserved group—high-risk antenatal patients—and the assessment of the full implementation of the Web-based intervention into hospital-based antenatal care. Specific research questions are:

1. What is the effectiveness of the integrated mental health intervention on (1) the prevalence and severity of prenatal depression, anxiety, and stress in antenatal inpatients and (2) the prevalence and severity of postnatal depression, anxiety, and stress at 3-months postpartum compared to preintervention?
2. What is the acceptability and feasibility of the intervention for women?
3. What is the logistical and economic feasibility of implementing the integrated mental health intervention as a component of routine hospital-based antenatal care? What is needed to improve the clinical utility of the intervention in the hospital setting?

### The Intervention

#### Rationale, Development, and Pilot Testing of the Intervention

The intervention was developed to address the need for prenatal mental health care in systems where assessment, referral, and treatment are not components of routine prenatal care. It was designed to (1) target the needs of pregnant women, recognizing that sources of anxiety and depression are unique among pregnant women; (2) overcome the most prominent barriers cited by women and health care providers regarding prenatal mental health screening and care (eg, lack of time, lack of knowledge regarding type and interpretation of screening tools, lack of linkages with mental health resources) [18,27,32,65]; and (3) provide an integrated system of assessment, referral, and treatment that would optimize the flow for providers and women from assessment to treatment. Developed by the Healthy Outcomes of Pregnancy and Postpartum Experiences (HOPE) Research Team for a pilot RCT (Integrated Maternal Psychosocial Assessment to Care Trial-Pilot; ClinicalTrials.gov identifier: NCT01901796) [43], the usability of the intervention was tested in a group of 8 pregnant women recruited from a survey-based study on views of prenatal mental health screening conducted by our team. Based on recommendations for the evaluation of health information systems [64,66,67], a research assistant met with participants individually in March 2013 and instructed them to “think aloud” as they interacted with the Web-based psychosocial assessment and CBT modules to describe their ease of use, esthetics, program capability, navigability, and content. Interviews were taped and transcribed verbatim and transcripts were reviewed by the research team to identify women’s recommendations. Minor changes were made to the CBT modules based on participants’ recommendations, primarily involving clarification of directions for exercises. Recruitment for the pilot trial will be completed by December 2014.

#### Description of the Intervention

The integrated mental health intervention is a Web-based intervention that is available through a password-protected Web link on a bedside computer terminal. The intervention consists of 3 components: (1) psychosocial assessment, (2) a clinical decision support system that uses results of the psychosocial assessment to generate a clinical risk score with a clinician prompt that guides the provider on the best referral/treatment approaches for that woman, and (3) CBT. Given the potential enhancement of treatment outcomes in Web-based CBT supplemented by supportive coaching [41], a nontherapeutic coach is assigned to each woman. The role of the coach is to (1) discuss psychosocial assessment results, discuss referral options, and set-up referrals; (2) contact women weekly via text-based messaging to encourage completion of the CBT modules and follow-up questionnaires; and (3) address general program or technical questions.

#### Psychosocial Assessment

On recruitment, women complete a single Web-based psychosocial assessment that combines a standardized screening tool (Edinburgh Postnatal Depression Scale, EPDS) to evaluate depression and anxiety symptoms in the past week with a holistic assessment of psychosocial risk factors, including mental health history, substance use, and interpersonal violence (Antenatal Risk Questionnaire, ANRQ-R) [3,28]. The ANRQ-R was designed to be embedded within an integrated system of assessment-referral-care to identify psychosocial risk factors associated with poor mental health outcomes in pregnant women. Both instruments can be completed in less than 10 minutes. The ANRQ-R has high levels of acceptability and satisfactory psychometric properties (sensitivity 0.62; specificity 0.64) [3,28], comparable to other commonly used self-report depression/anxiety tools. The EPDS is a widely used 10-item self-report depression scale used to detect depression symptoms during the previous 7 days [68]. Psychometrically validated for use in pregnant and postpartum women [69], testing revealed sound psychometric properties (sensitivity 86.7%; specificity 78%; positive predictive value 74%, α=.87) [68].

#### Web-Based Clinical Decision Support Systems

Using the EPDS and ANRQ-R scores, a Web-based decision algorithm automatically generates a clinical risk score that is linked to a clinician prompt describing the best referrals for that particular woman. Once women complete the EPDS and ANRQ, 1 of 10 clinical risk scores is calculated automatically based on the severity of symptoms and combination of risk factors (risk 1 highest to risk 10 lowest). As soon as women submit their data, they are transmitted to the Faculty of Medicine and Dentistry server. The clinical risk score and clinician prompt are then viewed by the coach who telephones each woman to discuss results of the psychosocial assessment and referral options and provides information on accessing the password-protected CBT modules. The decision support system was built for the pilot RCT [43] and has been pilot tested and refined. By way of example, for women who have mild or moderate symptoms of anxiety, stress, or depression, the Web-based CBT is the choice treatment. For women with severe symptoms, the clinical prompt would recommend Web-based CBT plus referral to a psychiatrist.

#### Web-Based Cognitive Behavioral Therapy Program

**Figure 1:**
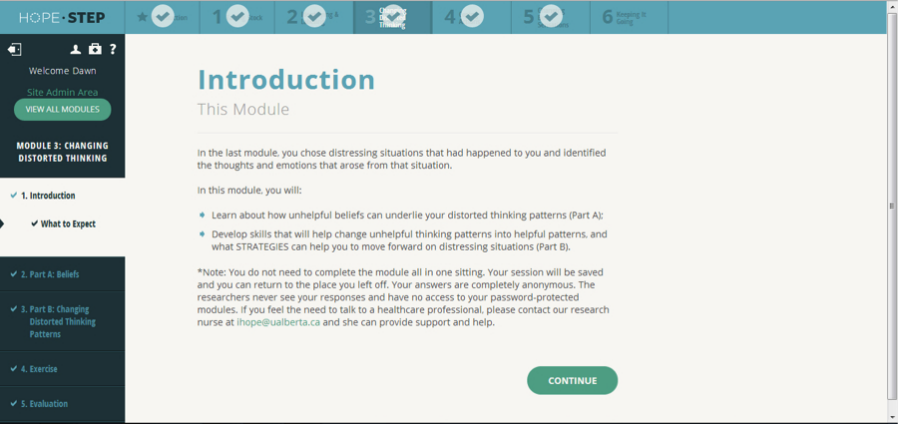
Screenshot of the introduction to the Web-based CBT module.

**Figure 2:**
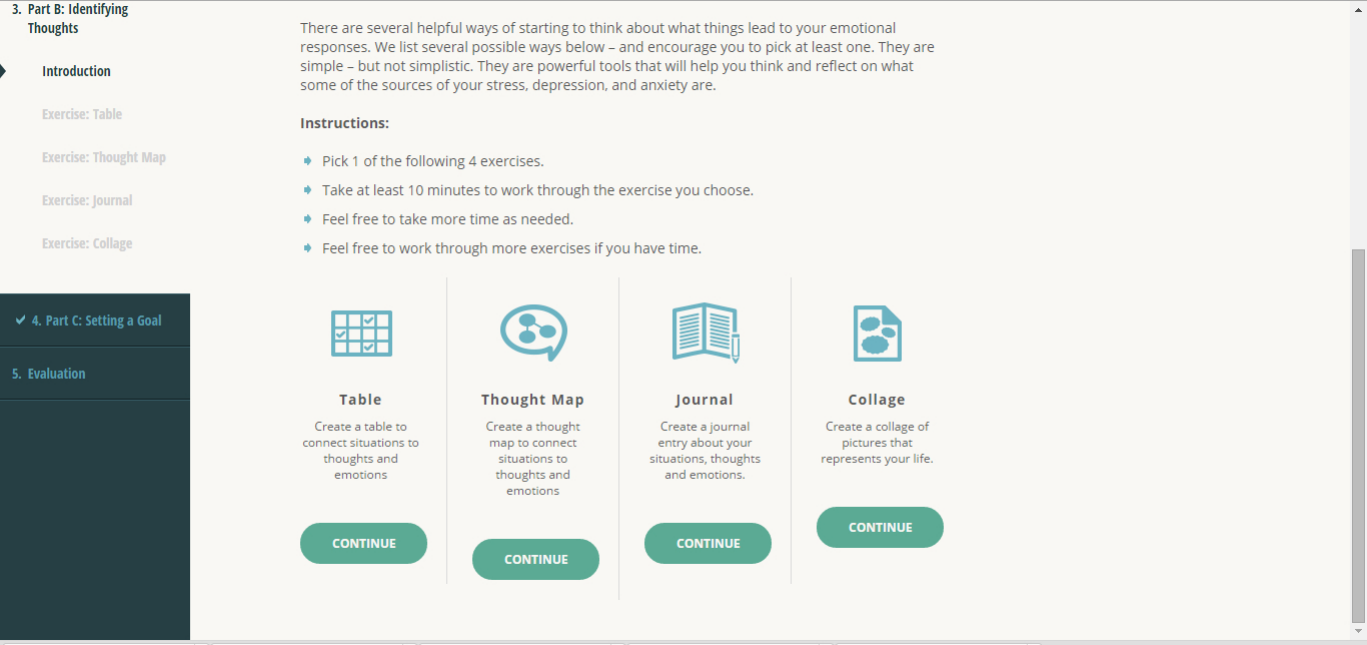
Screenshot of a sample exercise.

Women access the 6-module Web-based CBT program through a password-protected link. Two [70] to 6 [34] Web-based CBT sessions have been found to effectively reduce depression symptoms, and a recent feasibility study of Web-based CBT in postpartum women demonstrated completion rates of 87% in the 6-module program [34]. The topics of the modules are (1) taking stock; (2) identifying and labeling emotional health concerns; (3) changing distorted thinking; (4) understanding and changing actions, responses, and behavior; (5) relaxation; and (6) developing and maintaining a plan (Figures 1 and 2). Each module has interactive assignments that women complete. Each assignment has 1 to 4 options and women select the 1 (or more) that best suits their needs (Figure 2). Completion of the exercises is required before progression in the modules can occur. The modules use pregnancy-relevant scenarios and these are used as the basis of examples in the assignments. The Web-based delivery allows women to set their own pace by completing the modules at a time and location that is most convenient and ensures standardization of the intervention. Women access the modules using a username and password, and content that women provide in the assignments is accessible only by them.

## Methods

### Study Design

The proposed study is a before-after quasi-experimental design with a qualitative component. Because women in the antenatal unit interact frequently, it was not possible to avoid the contamination that would occur in a RCT. The study has 2 phases: (1) phase 1—the before-after study designed to evaluate the clinical- and cost-effectiveness of the integrated psychosocial assessment-referral-CBT intervention and (2) phase 2—a qualitative descriptive component designed to assess the utility, usability, feasibility, and acceptability of the intervention.

### Phase 1: Before-After Study

#### Setting and Recruitment Procedures

Recruitment will take place on a 24-bed antenatal inpatient unit at a tertiary care hospital in a large, urban Canadian city (Edmonton, Alberta). The hospital has more than 6500 annual births and draws patients from the northern half of the province to serve an ethnically and sociodemographically diverse population. The average length of stay on the unit is 5.5 days and the most common admission diagnoses are preterm labor, placenta previa, and hypertension.

#### Participant Eligibility and Recruitment Procedures

Pregnant women are eligible to participate if they are (1) <37 weeks gestation, (2) admitted to the antenatal inpatient unit for >72 hours, (3) able to speak and read English or be willing to use a translation service to assist with completion of the questionnaires and intervention, (4) able to complete follow-up email questionnaires, (5) aged >16 years, and 6) are not actively suicidal. Women admitted to the unit for induction (eg, <72-hour length of stay) are excluded.

Eligible women will be approached on admission by a research assistant who will describe the study and administer informed consent. Following consent, participants will complete a Web-based baseline questionnaire, which begins with the EPDS. Question 10 of the EPDS asks women about self-harm. For women who answer question 10 affirmatively, 4 additional questions pop-up to discriminate between suicidal ideation (ie, thinking about suicide with no plans) and active suicidality (ie, thinking about suicide with plans):

1. In the past week, have you sometimes felt hopeless about the future?
2. In the past week, have you sometimes wished you were dead?
3. In the past week, have you sometimes thought of ending your life?
4. If yes, is there anything that would stop you from acting on these thoughts of ending your life?

An affirmative response to any of these 4 questions would constitute active suicidality (=risk 1) and study exclusion. In this case, a computer message appears thanking the woman for her study participation and an email is sent to the research assistant immediately. Our safety protocol requires the research assistant to immediately inform unit nurses, who will arrange contact with hospital-based reproductive mental health services.

Women who remain eligible for the study following the EPDS completion will be permitted to continue with the baseline questionnaire for completion of the ANRQ-R and remaining baseline components. On submission, the data are securely stored in RedCap in the Faculty of Medicine and Dentistry at the University of Alberta. An automatic email informs the coach of the new participant. The coach accesses the woman’s psychosocial assessment results, the clinical risk score, and the clinician prompt in RedCap, and telephones the woman to discuss her results, referral options if applicable, and instructions for accessing the Web-based CBT. All coach contact is documented in a coach’s log in RedCap. One coach will be assigned to the recruitment site to ensure consistency across contacts.

#### Coach Training and Support

The coach will participate in a primary investigator-led 8-hour training course that addresses the structure of each of the 3 components of the intervention, study protocols (including arranging referrals), safety protocols, interpretation of assessment tools, approaches for describing assessment results, and managing follow-up. Processes are compiled in a Coach’s Guide that is provided during training. Didactic and scenario-based practice sessions will be used during the course. Weekly meetings with the primary investigator and monthly meetings with the broader research team will be used for troubleshooting and refinement of recruitment processes.

#### Sample Size Estimation and Feasibility

The sample size calculation is based on the primary outcome of symptoms of depression, anxiety, and stress as measured by the depression, anxiety, and stress subscales of the 21-item Depression Anxiety Stress Scale-21 (DASS21) [71]. We calculated the sample size required to test the minimum clinically important difference in each subscale and selected the highest 1 for the final sample size. Based on DASS21 data collected as part of Australia’s national perinatal mental health initiative, standard deviations for the depression, anxiety, and stress subscales in pregnant women are 5.4, 10.2, and 8.6 [72]. To determine the minimal clinically important difference, we used Milgrom et al’s [55] approach for calculating the difference in scores on each subscale that would shift a woman 1 level of severity—the minimal, reasonable expectation for an effective therapy. For example, the DASS21 manual “categorizes” women as having normal, mild, moderate, severe, and extremely severe symptoms of depression, anxiety, and stress [71]. To shift women from midrange moderate to mild severity on the depression, anxiety, and stress subscales would require a reduction of 4 points in each subscale. Therefore, based on the sample size formula for paired *t* tests (2-tailed) at a significance level of 5% (1.96), a power of 80% (.84), a minimal clinically important difference of 4 points, and standard deviations of 5.4, 10.2, and 8.6 for the depression, anxiety, and stress subscales, respectively, the number of women required to detect a statistically significant difference in pre- and posttest scores would be 17 for depression symptom changes, 54 for anxiety, and 39 for stress. Therefore, based on the highest number of women needed, 54 women are required to complete full data for this study. Accounting for a participation rate of 50% based on previous studies of CBT in pregnant women [73], a conservative attrition rate of 25% based on previous studies of prenatal CBT [34,64], and a 5% loss to follow-up, 98 women would need to be invited to participate in the study to achieve the final sample size. Given the estimated number of 20 new admissions per month, the duration of recruitment is anticipated to be 5 months.

#### Definition and Measurement of Outcomes

##### Primary Outcome

The primary outcome is the presence and severity of prenatal depression, anxiety, and stress symptoms at 6-8 weeks post-recruitment as measured by the DASS21 [71]. The DASS21 has been widely used and psychometrically tested, and it distinguishes well between symptoms of depression, anxiety, and stress in clinical and nonclinical populations [66,71,74]. It is used in clinical settings to screen pregnant and postpartum women for presence and severity of current symptoms of depression, anxiety, and stress [72,75]. The DASS21 has good psychometric properties with Cronbach alphas of .91, .80, and .84, respectively, for the depression, anxiety, and stress subscales [66]. High correlations with other standardized depression, stress, and anxiety measures (eg, Beck Depression Inventory, State-Trait Anxiety) and clinical assessments demonstrate its validity [76,77].

The presence of symptoms of prenatal depression, anxiety, and stress is measured as the proportion of women scoring above established DASS21 cut-offs (>10, >8, and >15, respectively) [71]. Severity of symptoms is measured by the mean depression, anxiety, and stress scores. Ranges of scores corresponding to symptom severity levels of normal, mild, moderate, and severe are also well established through psychometric testing: depression (normal: 0-9; mild: 10-13; moderate: 14-20; severe: >21), anxiety (normal: 0-7; mild: 8-9; moderate: 10-14; severe: >15), and stress (normal: 0-14; mild: 15-18; moderate: 19-25; severe: >26) [71].

##### Secondary Outcomes

The secondary clinical outcomes are presence and severity of symptoms of postpartum depression, anxiety, and stress [71]; prenatal and postnatal self-efficacy [78], social support [79], sense of mastery [80], self-esteem [81], sleep [82,83], relationship quality [10,84], coping [85], and resilience [86]; 5-minute Apgar score; gestational age; birth weight; maternal-infant attachment [87]; infant behavior [88]; infant development [86,89]; and parenting stress/competence [90,91]. These outcomes were selected because of their association with maternal depression, anxiety, and stress and their potential modifiability by the intervention. Table 1 presents the primary and secondary clinical outcomes.

**Table 1:** Measures (primary and secondary clinical outcomes; other) and timeline for phase 1 of the quasi-experimental study.

Measures		Timeline of assessments		
		Baseline	6-8 weeks	3-months postpartum
**Primary clinical outcome**				
	Prenatal depression, anxiety, stress symptoms (Depression Anxiety Stress Scale, DASS21) presence (% above cut-off point) and severity (mean score, SD)	X	X	
**Intervention component**				
	Psychosocial assessment (Antenatal Risk Questionnaire-Revised, ANRQ-R; includes substance use and violence)	X		
	Depression (Edinburgh Postnatal Depression Scale, EPDS)	X	X	X
**Secondary clinical outcome**				
	Postnatal depression, anxiety, stress symptoms (Depression Anxiety Stress Scale, DASS21) presence (% above cut-off point) and severity (mean score, SD)			X
	Social support (Interpersonal Support Evaluation List, ISEL)	X	X	X
	ANRQ-R acceptability	X		
	Mastery (Pearlin’s Mastery Scale)	X	X	X
	Self-efficacy (Generalized Self-Efficacy Scale)	X	X	X
	Self-esteem [81]	X	X	X
	Resilience (Connor-Davidson Resilience Scale)	X	X	X
	Sleep (Pittsburgh Sleep Quality Index)	X	X	X
	Parenting competence (Parenting Sense of Competence Scale, PSCS; subscales Efficacy, Interest, Satisfaction)			X
	Parenting stress (Parental Stress Scale)			X
	Relationship quality and adjustment (Dyadic Adjustment Scale, DAS-7)	X	X	X
	Coping (Brief Cope)	X	X	X
	Maternal-infant attachment [87]			X
	Infant behavior (Infant Behavior Questionnaire)			X
	Infant development (Ages and Stages Questionnaire, 3rd edition, ASQ-3; The Baby Pediatric Symptom Checklist for Social/Emotional Screening)			X
	Birthweight (medical record)			X
	Gestational age (medical record)			X
	5-minute Apgar score (medical record)			X
**Other**				
	Feeding method (medical record; parent report); neonatal/infant health (medical record; parent report) (Parent report from All Our Babies birth cohort study^a^)			X
	Demographics (education, income, maternal age at recruitment, ethnicity; items from Maternity Experiences Survey, MES^b^)	X		
	Obstetric and medical history (parity, chronic and pregnancy complications, type of delivery, weight at prepregnancy, delivery, 6 weeks postpartum) (self-report items from MES; medical record)	X		X
	Mental health history (history of depression, anxiety, stress; age of onset of previous episodes of mental health problems) (items from MES)	X		
	Pharmacologic therapy for depression/anxiety (past; current) (items from Canadian Community Health Survey, CCHS)	X	X	X

^a^ The All Our Babies Birth Cohort study is a pregnancy birth cohort in Alberta, Canada. Details of the study methodology and design have been previously published [92].

^b^ The Maternity Experiences Survey (MES) is a national survey designed and administered by the Public Health Agency of Canada and Statistics Canada [93].

Secondary process outcomes related to the overall feasibility of the intervention focus on its cost-effectiveness, efficiency, utility, usability, and acceptability (Table 2). We will evaluate the intervention feasibility from both the patients’ and providers’ perspectives using both quantitative (phase 1) and qualitative (phase 2) approaches. These data will be used to refine the intervention components to optimize their implementation into the hospital setting.

**Table 2:** Measures of secondary process outcomes.

Secondary process outcomes			Baseline	6-8 weeks	3 months postpartum
**Phase 1: quasi-experimental study**					
	**Cost-effectiveness**				
		Women’s health service use, medication use (self-report and medical record)	X	X	X
		Women’s quality of life (For economic analysis-SF-12,SF-6D to calculate QALY)	X	X	X
		Costs related to hospital-based implementation (eg, computer access; time to manage referrals)			X
	Efficiency of intervention (% of women with psychosocial assessment, referral, and care; self-report and medical record)		X	X	X
	Utility of intervention (1 question asked at the end of each CBT exercise: “This exercise was useful to me” with 4 response options of I strongly agree, I somewhat agree, I somewhat disagree, I strongly disagree; 1 question asked at the end of each CBT module: “The information in this module was useful to me” with same response options)		X	X	
	Usability of intervention (1 question asked at the end of each CBT exercise: “This exercise was clear and easy to understand” with response options; 2 questions asked at the end of each module: “The information in this module was clear and easy to understand” and “It was easy to work through the module [for example, it was easy for me to get from 1 part to the other, easy to find what I needed]” with same response options)		X	X	
	**Acceptability**				
		Web-based psychosocial assessment (1 question at end of completing ANRQ-R: “I would recommend a Web-based approach to asking about emotional health to a pregnant friend” with 4 response options of I strongly agree, I somewhat agree, I somewhat disagree, I strongly disagree)	X		
		CBT (1 question at end of each CBT module: “I would recommend this module to a pregnant friend who was struggling with stress, depression, or anxiety” with 4 response options of I strongly agree, I somewhat agree, I somewhat disagree, I strongly disagree)	X	X	
	Overall assessment (2 open-ended questions at the end of every CBT module: “The thing I liked most about this module was...” and “The thing I liked least about this module was...”)		X	X	
**Phase 2: qualitative descriptive study**					
	Efficiency (providers’ views of the efficiency of the intervention in facilitating referrals and care; women’s views on access to timely care)				X
	Utility (providers’ views on the usefulness of the intervention in promoting mental health assessment, providing guidance on referral/treatment; aiding referral process; women’s views of how useful the modules were in meeting their needs)				X
	Usability (women’s views of how easy/difficult the modules were to navigate)				X
	Feasibility (providers’ views of feasibility of the integrated intervention in their setting; women’s views of the feasibility of doing the modules; Google Analytics such as % women accessing CBT within 2 weeks postassessment; % women accessing each CBT module within 1-2 weeks; % completion of all 6 CBT modules; % completion of CBT modules within 8 weeks)				X
	Acceptability (providers’ views; women’s views)				X

#### Data Collection

##### Procedures

The 3 data collection points for all study participants are recruitment (pretest), 6-8 weeks postenrollment (posttest), and 3 months postpartum (Table 1). On recruitment, all consent and baseline data are completed on a link available on each patient’s bedside computer terminal. Follow-up questionnaires will be completed online. Participants will receive an email with a password and link to the Web-based questionnaire. Retention will be enhanced using Dillman’s approach [94] in which women who have not completed the questionnaires within 1 week will receive automated email/mobile phone reminders at 1, 3, 7, 10, and 14 weeks by RedCap. We will track reasons for nonadherence (eg, lost to follow-up).

##### Data Management

No data are stored on the bedside computers; when women submit their information, it is sent to a secure server housed in the Faculty of Medicine and Dentistry’s Data Centre (University of Alberta). Data transfer between the computer and server is encrypted. Follow-up questionnaires will be distributed and submitted via email that is also encrypted. All processes involving electronic data capture and storage are managed by the Women’s and Children’s Health Research Institute Informatics Core at the University of Alberta. Once recruitment has been completed, the Informatics Core will transfer data to the Health Research Data Repository at University of Alberta. The Repository is a secure, interactive environment offering storage and interactive platforms for data analysis. Electronic data will be stored for 5 years at the Data Centre and then deleted. Research team members requiring direct access to data will complete a confidentiality orientation by the Repository Manager.

##### Adherence, Fidelity, and Concomitant Care

Adherence to the intervention will be tracked through Google Analytics and analytics designed for this study (eg, number modules completed, length of time to complete modules, etc). As part of the qualitative descriptive component, we will seek women’s opinions about aspects of the psychosocial assessment that were challenging and features of the CBT modules that affected their ability, need, or desire to complete them. To improve adherence, the coach will send weekly text messages to women describing the importance of regular progress through the module exercises, the benefit of completing all modules, and encouragement when modules are completed. In addition, an automatic email or text message reminder will be generated if women have been inactive on the site for more than 2 weeks. The Web-based format of the intervention preserves its fidelity (ie, consistency in its components and delivery) and thus enhances external validity. To account for cointervention, follow-up questionnaires will ask women to disclose any pharmacological or nonpharmacological therapy that they have begun and this additional intervention will be accounted for in the analyses.

#### Ethics Considerations

The study protocol was approved by the Human Research Ethics Board at the University of Alberta. Following electronic consent, all women receive an emailed copy of the Participant Information Letter and Consent.

#### Safety Protocol

Several strategies ensure women’s safety throughout the study. Mental health crisis contact information is described on a sidebar of the CBT modules, along with a statement encouraging women to contact their coach if they feel their mental health is deteriorating. The coach will contact women within 24 hours using a defined algorithm to guide decisions regarding help or referral that is recommended.

At the end of each CBT module, women will complete question 10 of the EPDS to assess self-harm thoughts over the past week. An affirmative response will generate an automatic message with crisis contact information for the woman’s immediate use and an email sent to the coach. The coach will contact the woman within 24 hours to assess whether the woman is receiving help from a health care provider. A 4% affirmative response rate to question 10 of the EPDS has been reported [14]. The coach will document all interactions in the coach’s log.

#### Analyses

##### Effectiveness of Intervention

We will use descriptive data (frequencies and 95% CIs; means and SDs) for sample description. We will assess differences in pre- and posttest means using paired *t* tests and proportions using McNemar tests. We will generate multivariable logistic regression models to identify predictors of intervention success, reporting relative risks and 95% CIs. Multivariable regression models will be built using variables that are associated with outcomes at *P*<.10 on unadjusted analyses. Primary analyses will use a type I error of 5% as a criterion for statistical significance, whereas a more stringent alpha of .01 will be used for secondary outcomes to account for multiple testing. Because women will be starting the intervention at different points in pregnancy, we will control for gestation. We will conduct exploratory analyses using stratified analyses to explore differences of intervention effect by (1) number CBT modules completed, (2) antidepressant or use of nonpharmacological therapy, (3) severity of DASS21 scores, (4) participant characteristics, (5) mental health history, and (6) gestational age. We do not plan to do imputation of missing data because we anticipate that the Web-based questionnaires with required fields will result in a low percentage of missing data.

##### Efficiency, Utility, Usability, and Acceptability of Intervention

In addition to assessing efficiency, utility, usability, and acceptability of the intervention through qualitative interviews (phase 2), we will use descriptive statistics (frequencies, proportions, means, SDs) to describe the efficiency of the intervention (eg, percentage of women with psychosocial assessment, referral, and care pretest vs posttest) and women’s perceptions of the intervention’s utility (eg, rated usefulness of exercises and information), usability (eg, ease of exercises and module), and acceptability (completion rates, willingness to recommend intervention to a friend).

#### Cost-Effectiveness of Hospital-Based Intervention

The economic evaluation will be a within-study cost-effectiveness analysis comparing the intervention with usual hospital-based mental health care. The analysis will assess costs associated with the delivery of the intervention (eg, cost of equipment, salary of coach) and subsequent service utilization by study participants. Direct health care utilization will be extracted from patient records. Data related to health and social care utilization will be collected from the medical record and self-reported by women (including SF-12). The primary outcome measure for the cost-effectiveness analysis will be the Quality Adjusted Life Year (QALY). Utilities for the construction of QALYs will be obtained from the SF-12 data using the SF-6D algorithm [95]. Because the time horizon for the analysis is less than 12 months, discounting will not be required [96]. We will report the incremental cost per QALY gained for the intervention compared to usual prenatal care. Uncertainty in the expected costs and outcomes for the integrated intervention and usual prenatal care will be characterized using the nonparametric bootstrap. The results of the bootstrap analysis will be used to construct scatterplots on the cost-effectiveness plane and cost-effectiveness acceptability curves showing the probability that the integrated intervention is a cost-effective use of health care resources for a range of values of health.

### Phase 2: Qualitative Descriptive Study to Assess Overall Feasibility of the Intervention

#### Design and Rationale

Phase 2 is a qualitative descriptive study with a primary aim of assessing women’s and health care providers’ views on efficiency, utility, usability, feasibility, and acceptability of the intervention. Phase 2 is a critical component to support further refinement of the intervention that will optimize its feasibility for women and providers, and enhance women’s engagement and adherence [58].

#### Participant Eligibility and Recruitment

All women and health care providers working at the study site are eligible for participation in phase 2. Purposeful sampling will be used to maximize variability in the sample, ensuring that a broad range of views and demographics are represented [97]. We plan to interview 15-20 women and 10-15 providers (eg, unit staff, executive director, managers, reproductive mental health service staff, physicians) with the final sample size established by data saturation. Given the importance of understanding factors contributing to attrition, we will also interview women who do not complete all CBT modules. To capture these women, a statement at the end of each of the final 3 CBT modules will invite women to participate in a follow-up interview. Selection of the affirmative response will generate an automatic email to the research coordinator for follow-up. Posters and staff meetings will be used to invite unit staff members to participate in a follow-up interview.

#### Data Collection and Management

We will conduct individual face-to-face or telephone-based interviews. Semistructured interview guides will be used [97] to ask participants their views on the efficiency, utility, usability, feasibility, and acceptability, as well as its strengths, suggestions for improvement, components that were effective/not effective, and the benefits that they experienced. The anticipated length of the interviews is 30 minutes. They will be digitally recorded and transcribed verbatim. Transcribed interviews and digital files will be stored in the Health Research Data Repository (University of Alberta) and stored for 5 years. All data will be anonymized for publication.

#### Analysis

We will use standard qualitative content analysis approaches for thematic analysis [97]. Two members of the team experienced in qualitative analysis will independently code the first 2 or 3 transcripts and engage in discussion to reach consensus on a draft coding scheme. This coding scheme will be used to code 2 additional transcripts with revisions made as necessary. Subsequent transcripts will be coded by 1 team member. Analysis will occur concurrently with data collection to allow further exploration and clarification of emergent ideas, and data collection will continue until data saturation [98].

## Results

The study was funded in September, 2014 and ethics was approved in November, 2014. Subject recruitment will begin January, 2015 and results are expected in December, 2015. Results of this study will determine (1) the effectiveness of an integrated Web-based prenatal mental health intervention on maternal and infant outcomes and (2) the feasibility of implementation of the intervention on a high-risk antenatal unit.

## Discussion

Results of this feasibility study will guide the refinement of the 3 components of the Web-based mental health intervention and full integration in the hospital setting. In this study, the research coach plays the role of coach/case manager in that she maintains regular supportive contact with participants, reviews women’s psychosocial assessment results and debriefs them, and organizes referrals as well as linkage to the Web-based CBT program. The next steps would involve hospital-based personnel adopting this role and integration of the Web-based assessment and clinical decision support system into the electronic medical record.

## Multimedia Appendix 1

Decision letter (grant review - CIHR).

## Abbreviations

CBT: cognitive behavioral therapy
DASS21: 21-item Depression Anxiety Stress Scale
EPDS: Edinburgh Postnatal Depression Scale
HOPE: Healthy Outcomes of Pregnancy and Postpartum Experiences
MES: Maternity Experiences Survey
QALY: Quality Adjusted Life Year
RCT: randomized controlled trial
