# Supplementary material for: An Integrated Web-Based Mental Health Intervention of Assessment-Referral-Care to Reduce Stress, Anxiety, and Depression in Hospitalized Pregnant Women With Medically High-Risk Pregnancies: A Feasibility Study Protocol of Hospital-Based Implementation
Source: JMIR Res Protoc. 2015 Jan 16;4(1):e9. doi: 10.2196/resprot.4037 (PMC4319085; doi:10.2196/resprot.4037)
Supplement: Supplementary file 1 [file resprot_v4i1e9_app1.pdf]

Canadian Institutes of Health Research/Instituts de recherche en santé du Canada

Notice of Recommendation/Avis de recommandation

Application Number/Numéro de la demande: 287111
Committee Code/Code du comité: HS2

Applicants/Candidats: Dr. Dawn Aileen KINGSTON

With/Avec:

Prof. M. AUSTIN	Dr. A. BIRINGER	Dr. K. HEGADOREN
Ms. G. LASIUK	Dr. G. MACQUEEN	Dr. S. MCDONALD
Dr. P. MCGRATH	Dr. D. SCHOPFLOCHER	Mrs. S. SPRENG
Dr. W. SWORD	Dr. S. VELDHUYZEN VAN ZANTEN	

Institution paid/ Établissement payé: University of Alberta

Title/Titre: Integrated Maternal Psychosocial Assessment to Care Trial (IMPACT): Intervening Early to Improve Maternal and Child Health

Primary Inst./Inst. principal: Health Services and Policy Research
Other Related Inst./ Autres inst. connexes: Human Development, Child and Youth Health

Competition /Concours: Operating Grant
September/Septembre 17, 2012

Number in competition/Nbre de demandes dans le concours: 2333

Peer Review Committee Recommendation, for your information and use/
Recommandation du comité d'examen par les pairs, pour fins d'information et d'utilisation:

**Committee/Comité:**	Health Services Evaluation & Interventions Research 2
**Number reviewed/ Demandes examinées:**	53
**Application rank within the committee/ Rang de la demande dans le comité:**	4
**Percent Rank within the committee / Rang en pourcentage au sein du comité:**	7.55%
**Rated / Cote:**	4.37
**Recommended Term/ Durée recommandée:**	4 years/ans 0 months/mois
**Recommended average annual operating amount/ Montant annuel moyen recommandé pour le fonctionnement:**	$113,892
**Recommended equipment amount/ Montant recommandé pour les appareils:**	$0

This document is for information only.
An application rated below 3.50 is ineligible for CIHR funding. For applications rated 3.50 and above, please note that it is the application's rank within the peer review committee that determines whether it is funded, rather than its absolute rating. The final funding decision will be communicated in the Notice of Decision.

Document à titre d'information seulement.
Une demande cotée en dessous de 3,5 n'est pas admissible au financement des IRSC. En ce qui a trait aux demandes cotées 3,50 ou plus, veuillez noter que l'on détermine l'attribution des fonds en fonction du classement obtenu au sein du comité d'examen par les pairs plutôt qu'en fonction du classement absolu. La décision finale relative au financement sera communiquée dans l'Avis de décision.

January/Janvier 24, 2013

DC0190GP

Canadian Institutes of Health Research / Instituts de recherche en santé du Canada

Notice of Decision / Avis de décision

Application Number/Numéro de la demande: 287111

Committee Code/Code du comité: HS2

Applicants/Candidats: Dr. Dawn Aileen KINGSTON

With/Avec:				
	Prof. M. AUSTIN	Dr. A. BIRINGER	Dr. K. HEGADOREN	Ms. G. LASIUK
	Dr. G. MACQUEEN	Dr. S. MCDONALD	Dr. P. MCGRATH	Dr. D. SCHOPFLOCHER
	Mrs. S. SPRENG	Dr. W. SWORD	Dr. S. VELDHUYZEN VAN ZANTEN	

Institution paid/ Établissement payé: University of Alberta

Title/Titre: Integrated Maternal Psychosocial Assessment to Care Trial (IMPACT): Intervening Early to Improve Maternal and Child Health

Primary Inst./ Inst. principal: Health Services and Policy Research

Other Related Inst./ Autres inst. connexes: Human Development, Child and Youth Health

**Competition Outcome/Résultats du concours:**	Operating Grant<br>September/Septembre 17, 2012
**Number in competition/Nbre de demandes dans le concours:**	2333
**Number approved/Nbre de demandes approuvées:**	401

**Decision on your application/ Décision sur votre demande:**	Approved
**Average annual amount/ Montant annuel moyen:**	$86,558
**Equipment amount/ Montant pour les appareils:**	$0
**Term/Durée:**	**4 yrs/ans 0 months/mois**

Peer Review Committee Recommendation, for your information and use/ Recommandation du comité d'examen par les pairs, pour fins d'information et d'utilisation:

**Committee/Comité:**	Health Services Evaluation & Interventions Research 2
**Number reviewed/ Nbre de demandes examinées:**	53
**Number approved in that committee/ Nbre de demandes approuvées dans ce comité:**	10
**Application rank within the committee/ Rang de la demande dans ce comité:**	4
**Percent Rank Within the Committee/ Rang en pourcentage au sein du comité:**	7.55%
**Rating/ Cote:**	4.37
**Recommended average annual amount/ Montant annuel moyen recommandé:**	$113,892
**Recommended equipment amount/ Montant recommandé pour les appareils:**	$0

**Additional Funding Opportunities/ Opportunités de financement additionnelles**	**Decision/ Décision**	**Competition Code/ Cote de concours**	**Application Number/ Numéro de la demande**
Operating Grant - PA: Reproductive and Child Heath (start-up grants)	Not Approved	201209IHD	295570

*** Applications receiving a score of less than 3.5 on any evaluation criteria will not be considered for Funding. / Les demandes qui ont reçu une note inférieure à 3.5 pour n'importe quel des critères d'évaluation ne sont pas admissibles.

CIHR IRSC

Institute of Aboriginal Peoples' Health
Institute of Aging
Institute of Cancer Research
Institute of Circulatory and Respiratory Health
Institute of Gender and Health
Institute of Genetics
Institute of Health Services and Policy Research
Institute of Human Development and Child and Youth Health
Institute of Infection and Immunity
Institute of Musculoskeletal Health and Arthritis
Institute of Neurosciences, Mental Health and Addiction
Institute of Nutrition, Metabolism and Diabetes
Institute of Population and Public Health

Institut de la santé des Autochtones
Institut du vieillissement
Institut du cancer
Institut de la santé circulatoire et respiratoire
Institut de la santé des femmes et des hommes
Institut de génétique
Institut des services et des politiques de la santé
Institut du développement et de la santé des enfants et des adolescents
Institut des maladies infectieuses et immunitaires
Institut de l'appareil locomoteur et de l'arthrite
Institut des neurosciences, de la santé mentale et des toxicomanies
Institut de la nutrition, du métabolisme et du diabète
Institut de la santé publique et des populations

January 24, 2013

Dr. Dawn Aileen KINGSTON
5-258 Edmonton Clinic Health Academy
University of Alberta
11405-87th Ave
Edmonton, Alberta T6G 1C9

Dear Dr. KINGSTON:

We are pleased to inform you that the Canadian Institutes of Health Research (CIHR) has approved your recent application entitled "Integrated Maternal Psychosocial Assessment to Care Trial (IMPACT): Intervening Early to Improve Maternal and Child Health". If you are receiving this letter through ResearchNet, your Authorization for Funding will follow in the mail otherwise it is enclosed in this package.

If you have not already received the review documents related to your proposal, please contact us. Should you have any questions about the review process, please address them directly to CIHR staff. Do not contact the officers or members of the peer review committee. As CIHR does not notify co-applicants of the decision, we ask that you inform those individuals involved, along with their research institutions (if different from your own), of the outcome of this application.

Congratulations on your success in this competition.

Gregory Huyer, Ph.D.
Deputy Director, Open Programs
Research and Knowledge Transition Portfolio

316765-201209MOP-HS2-287111-149863-DLGAR

Canadian Institutes of Health Research
Room 97, 160 Elgin Steet, Address locator: 4809A
Ottawa, (Ontario) K1A 0W9 Tel.: (613) 941-2672
Fax (613) 954-1800 **www.cihr-irsc.gc.ca**

Instituts de recherche en santé du Canada
Pièce 97, 160 rue Elgin, Indice de l'adresse: 4809A
Ottawa, (Ontario) K1A 0W9 Tél.: (613) 941-2672
Fax (613) 954-1800 **www.irsc-cihr.gc.ca**

Canada

CIHR IRSC

Institute of Aboriginal Peoples' Health

Institute of Aging

Institute of Cancer Research

Institute of Circulatory and Respiratory Health

Institute of Gender and Health

Institute of Genetics

Institute of Health Services and Policy Research

Institute of Human Development and Child and Youth Health

Institute of Infection and Immunity

Institute of Musculoskeletal Health and Arthritis

Institute of Neurosciences, Mental Health and Addiction

Institute of Nutrition, Metabolism and Diabetes

Institute of Population and Public Health

Institut de la santé des Autochtones

Institut du vieillissement

Institut du cancer

Institut de la santé circulatoire et respiratoire

Institut de la santé des femmes et des hommes

Institut de génétique

Institut des services et des politiques de la santé

Institut du développement et de la santé des enfants et des adolescents

Institut des maladies infectieuses et immunitaires

Institut de l'appareil locomoteur et de l'arthrite

Institut des neurosciences, de la santé mentale et des toxicomanies

Institut de la nutrition, du métabolisme et du diabète

Institut de la santé publique et des populations

January 24, 2013

Dr. Dawn Aileen KINGSTON
5-258 Edmonton Clinic Health Academy
University of Alberta
11405-87th Ave
Edmonton, Alberta T6G 1C9

Dear Dr. KINGSTON:

Congratulations on your success in the recent Canadian Institutes of Health Research funding competition.

You should take great pride in your success, particularly in light of the very competitive nature of CIHR peer review.

As you know, peer review is the cornerstone of our research funding system. This process rests on the kind voluntarism of your colleagues at other institutions who generously gave their time to review your application.

The Canadian Institutes of Health Research is committed to building an innovative national health research enterprise. To this end we have initiated a process to design a new Open Suite of Programs and peer review system to ensure the long-term sustainability of CIHR's contribution to the Canadian health research enterprise, to remove barriers, and to enable researchers from all pillars to improve CIHR's ability to deliver on its mandate.

To meet CIHR goals, we must also continue to communicate the value of health research to Canadians. That is why we encourage you to work with your institution to promote your research . We have developed guidelines on public communication, available on our website at http://www.cihr-irsc.gc.ca/e/30789.html, to support you in this activity.

Once again, congratulations and I wish you success in your research.

Yours sincerely,

Alain Beaudet, MD, Ph.D.
President

316780-201209MOP-HS2-287111-149863-CONGR

President

Canadian Institutes of Health Research
Room 97, 160 Elgin Steet, Address locator: 4809A
Ottawa, (Ontario) K1A 0W9 Tel.: (613) 941-2672
Fax (613) 954-1800 **www.cihr-irsc.gc.ca**

Président

Instituts de recherche en santé du Canada
Pièce 97, 160 rue Elgin, Indice de l'adresse: 4809A
Ottawa, (Ontario) K1A 0W9 Tél.: (613) 941-2672
Fax (613) 954-1800 **www.irsc-cihr.gc.ca**

Canada

Review Type/Type dévaluation: Committee Member 1/Membre de comité 1
Name of Applicant/Nom du chercheur: KINGSTON, Dawn Aileen
Application No./Numéro de demande: 287111
Agency/Agence: CIHR/IRSC
Competition/Concours: 2012-09-17 Operating Grant/Subvention de fonctionnement
Committee/Comité: Health Services Evaluation & Interventions Research 2/Recherche en interventions et en évaluation dans les services de santé 2
Title/Titre: Integrated Maternal Psychosocial Assessment to Care Trial (IMPACT): Intervening Early to Improve Maternal and Child Health

Assessment/Évaluation:

Application 287111 KINGSTON

1. *Synopsis* 'Integrated Maternal Psychosocial Assessment to Care Trial (IMPACT): Intervening Early to Improve Maternal and Child Health':

o *purpose of the proposal*: to evaluate the acceptability, clinical- and cost-effectiveness of a sustainable, innovative, integrated process of **online psychosocial assessment, referral, and cognitive behaviour therapy (CBT)** for pregnant women – focused on depression and anxiety. Intervention is an online CBT program comprising 6, 30-minute modules in 272 pregnant women prior to 32 weeks gestation at non-high risk for depression, anxiety, stress

o *hypothesis to be tested, or the research questions to be answered*: early interventioncan improve maternal mental health in pregnancy/postpartum and should improve stress outcomes

o *objectives to be achieved and approach proposed*: in a randomized controlled trial and interviews, main outcome is reduced symptoms of prenatal depression/anxiety/stress (minimal clinically important difference of 4 points in each of the depression, anxiety, and stress subscales of the DASS21). Secondary clinical outcomes are reduced risk of poor child (birth weight, Apgar scores), maternal (postpartum depression/anxiety/stress, parenting competence, parenting stress, maternal-child attachment, sleep quality) and family outcomes (partner relationship, maternal-child attachment). There is a prospective economic evaluation, i.e. a within-trial cost effectiveness analysis comparing the integrated intervention 'package' with usual prenatal care. The perspective of the primary economic evaluation will be that of the health and social care budget; a secondary analysis will adopt a societal perspective incorporating personal costs and productivity costs in addition to the health and social costs associated with the delivery of the intervention and subsequent service utilization by study participants. Project has 2 phases: (1) evaluation of the integrated psychosocial assessment-referral-CBT intervention (RCT); and (2) assessment of the feasibility, acceptability, and mechanisms of the intervention (qualitative interviews).

o *progress made to date*: findings of a pilot for his IMPACT-trial revealed that women found the program acceptable with recommendations that it be offered: (1) *earlier* in pregnancy; (2) in an online format; and (3) as shorter modules while retaining the content

2. *Assessment of the proposal*

**Criterion #1: Research Approach**	**Application**
· Clarity of the research question.	Clear: "What is the cost-effectiveness of an integrated prenatal psychosocial assessment, referral, and care on maternal and infant/child outcomes and what are the mechanisms behind the effect of the prenatal intervention?"
· Completeness of the literature review and relevance to study design/research plan.	· Sufficiently complete
· Clarity of rationale for the research approach and methodology.	· Clearly a clinical trial with economic evaluation is needed to answer the research question
· Appropriateness of the research design.	· Appropriate, see comment below re. risk of bias
· Appropriateness of the research methods.	· Appropriate, validated scales are used
· Feasibility of the research approach (including recruitment of	· Seems all feasible

Review Type/Type dévaluation: Committee Member 1/Membre de comité 1
Name of Applicant/Nom du chercheur: KINGSTON, Dawn Aileen
Application No./Numéro de demande: 287111
Agency/Agence: CIHR/IRSC
Competition/Concours: 2012-09-17 Operating Grant/Subvention de fonctionnement
Committee/Comité: Health Services Evaluation & Interventions Research 2/Recherche en interventions et en évaluation dans les services de santé 2
Title/Titre: Integrated Maternal Psychosocial Assessment to Care Trial (IMPACT): Intervening Early to Improve Maternal and Child Health

Assessment/Évaluation:

subjects, project timeline, preliminary data where appropriate, etc.).	
· Anticipation of difficulties that may be encountered in the research and plans for management.	· This is an evaluation of a complex intervention, and the intervention is unblended, and outcome is self reported… Good address of potential sources of bias, though, need to prevent spill-over of information and expectations with women in the control group
**Criterion #2: Originality of the Proposal**	
· Potential for the creation of new knowledge.	· Good potential, as there are no studies that evaluate an integrated prenatal psychosocial assessment-referral-care intervention, and there is only one trial that has evaluated the effect of prenatal psychosocial care on child outcomes
· Originality of the proposed research, in terms of the hypotheses/research questions addressed, novel technology/methodology, and/or novel applications of current technology/methodology.	· Non-Original hypotheses, research questions addressed, application of current technology & methodology.<br>· Yet, original hypotheses, research questions addressed, novel technology & methodology.
**Criterion #3: Applicant(s)**	
· Qualifications of the applicant(s), including training, experience and independence (relative to career stage).	· PI is assistant professor in nursing and in obs/gyn, well trained, experienced. Certainly qualified for this project
· Experience of the applicant(s) in the proposed area of research and with the proposed methodology.	· P12, Table 3. Sufficient experience in trial and survey methodology and management; quantitative analysis, CEA, content/clinical expertise
· Expertise of the applicant(s), as demonstrated by scientific productivity over the past five years (publications, books, grants held, etc.). Productivity should be considered in the context of the norms for the research area, applicant experience and total research funding of the applicant.	· Sufficient expertise and experience for this project
· Ability to successfully and appropriately disseminate research findings, as demonstrated by knowledge translation activities (publications, conference presentations, briefings, media engagements, etc.).	· Sufficient previous knowledge translation activities
· Appropriateness of the team of applicants (if more than one applicant) to carry out the proposed research, in terms of complementarity of expertise and synergistic potential.	· Nice complementarity of expertise and synergistic potential in this team

Review Type/Type dévaluation: Committee Member 1/Membre de comité 1
Name of Applicant/Nom du chercheur: KINGSTON, Dawn Aileen
Application No./Numéro de demande: 287111
Agency/Agence: CIHR/IRSC
Competition/Concours: 2012-09-17 Operating Grant/Subvention de fonctionnement
Committee/Comité: Health Services Evaluation & Interventions Research 2/Recherche en interventions et en évaluation dans les services de santé 2
Title/Titre: Integrated Maternal Psychosocial Assessment to Care Trial (IMPACT): Intervening Early to Improve Maternal and Child Health

Assessment/Évaluation:

**Criterion #4: Environment for the Research**	
· Availability and accessibility of personnel, facilities and infrastructure required to conduct the research.	· Sufficient for this project
· Suitability of the environment to conduct the proposed research.	· Suitable for this project
· Suitability of the environment (milieu, project and mentors) for the training of personnel (if applicable).	· Suitable mentors for the training of this project's personnel
**Criterion #5: Impact of the Research**	
· Research proposal addresses a significant need or gap in health research and/or the health care system.	· Proposal does addresses a significant gap in health research: randomized evidence of early diagnosis and intervention in pregnant women to enhance perinatal mental health
· Potential for a significant contribution to the improvement of people's health in Canada and the world and/or to the development of more effective health services and products.	· If proven effective, this feasible, sustainable, and accessible intervention can be widely implemented in primary care to improve maternal prenatal and perinatal mental health that will impact family well-being, parenting, and child mental health and development. In that case, good potential to lead to more effective health services and products
· Appropriateness and adequacy of the proposed plan for knowledge dissemination and exchange	· Research team has high degree of expertise and success in translating research findings to policy at provincial and national levels. Adequate plan for knowledge dissemination and exchange, including engaging end-users' interpretations on the findings and decision makers, (Provincial Task Group and Medical Officer of Health).

3. Budget requested

Seems appropriate.

4. Issues that should be flagged

None.

Review Type/Type dévaluation: Committee Member 2/Membre de comité 2
Name of Applicant/Nom du chercheur: KINGSTON, Dawn Aileen
Application No./Numéro de demande: 287111
Agency/Agence: CIHR/IRSC
Competition/Concours: 2012-09-17 Operating Grant/Subvention de fonctionnement
Committee/Comité: Health Services Evaluation & Interventions Research 2/Recherche en interventions et en évaluation dans les services de santé 2
Title/Titre: Integrated Maternal Psychosocial Assessment to Care Trial (IMPACT): Intervening Early to Improve Maternal and Child Health

Assessment/Évaluation:

Synopsis

The overall research question in this grant is "Does a prenatal cognitive behavioral care program reduce maternal stress and improve maternal and infant outcomes?" The applicants stress this project is about assessing integrated care of assessment-referral-treatment. In brief, assessment refers to using a screening tool to assess risk. High-risk mothers are referred to family physician, low-moderate risk mothers are referred to cognitive behavioral therapy (CBT) and no-risk mothers are provided their results and followed up but no treatment provided.

The intervention "group CBT" is reported effective in pregnant and new mothers. However, resources are limited. The applicants argue that mothers with low-moderate symptoms are unable to access CBT and are a vulnerable population and need to be able to access CBT on their own. Therefore, this grant proposes to develop an internet-based version of a successful CBT program that is delivered in 6 30-min modules over 4-6 weeks.

Strengths and Weaknesses

The applicants are using an RCT design to evaluate an intervention on an important clinical question, and supplementing the quantitative analysis with qualitative interviews to determine acceptability of the intervention. The internet-based intervention has the potential to reach a much larger population than is achieved with current methods. One weakness of this study is that the vulnerable population is being defined based on scores from a screening tool. These tools were specifically developed for screening – cut offs for scores should not be considered diagnostic. Therefore, I would prefer more evidence than what is currently in the grant about how "vulnerable" the proposed vulnerable population truly is. The analysis section could be clearer. The subgroup analyses appear to include post-randomization variables in the regression model. This would be appropriate for decomposing total causal effects into direct and indirect, but it appears the applicants are planning to interpret the statistical model as a total causal effect.

Research Question

Does a prenatal cognitive behavioral care program reduce maternal stress and improve maternal and infant outcomes? More specifically, the applicants want to determine clinical effectiveness of their internet-based

Review Type/Type dévaluation: Committee Member 2/Membre de comité 2
Name of Applicant/Nom du chercheur: KINGSTON, Dawn Aileen
Application No./Numéro de demande: 287111
Agency/Agence: CIHR/IRSC
Competition/Concours: 2012-09-17 Operating Grant/Subvention de fonctionnement
Committee/Comité: Health Services Evaluation & Interventions Research 2/Recherche en interventions et en évaluation dans les services de santé 2
Title/Titre: Integrated Maternal Psychosocial Assessment to Care Trial (IMPACT): Intervening Early to Improve Maternal and Child Health

---

Assessment/Évaluation:

CBT program, evaluate if integrated psychosocial care is efficient and feasible and cost-effective, and determine mediators and moderators of the intervention effect.

Literature Review

The applicants suggest that depression, anxiety and stress are under detected, and many women never seek treatment. The applicants argue that because symptoms begin during pregnancy, effective treatment is necessary at that time but do not provide any evidence of previous treatments.

Research Design

This is an RCT that includes qualitative interviews at a later date to determine feasibility, acceptability and mechanisms.

Research Methods

The inclusion criteria are pregnant women between 12-22 weeks gestation. Women with no stress are included because they could develop stress later. Women at high risk will be analyzed but treatment is referral to family physician (4-10% of subjects).

Mothers are recruited from one maternity clinic with 5 family physicians. First eligibility survey completed online with tablet in office. The applicants propose to use psychosocial assessment using screening tools (Edinburgh Postnatal Depression Scale (EPDS) and Antenatal Risk Questionnaire (ANRQ)). One limitation is that these are screening tools. Although many publications interpret them as clinical measures of stress and depression, they are not.

The CBT program itself had been pilot tested in paper form and adapted based on feedback in a pilot study. However, this intervention is an online version of the program, involving 6 30-min modules to be completed over 4-6 weeks. The online version is not yet developed. The control group gets usual care.

Outcomes of stress will be measured at 6-8 weeks post-randomization and 12 weeks postpartum: maternal stress, self-efficacy, self-esteem, sleep, and coping skills. Outcome measures are the 21-item Depression Anxiety Stress Scales (DASS21) which has been categorized into no, mild, moderate and severe scores based

**Review Type/Type dévaluation:**	Committee Member 2/Membre de comité 2
**Name of Applicant/Nom du chercheur:**	KINGSTON, Dawn Aileen
**Application No./Numéro de demande:**	287111
**Agency/Agence:**	CIHR/IRSC
**Competition/Concours:**	2012-09-17 Operating Grant/Subvention de fonctionnement
**Committee/Comité:**	Health Services Evaluation & Interventions Research 2/Recherche en interventions et en évaluation dans les services de santé 2
**Title/Titre:**	Integrated Maternal Psychosocial Assessment to Care Trial (IMPACT): Intervening Early to Improve Maternal and Child Health

Assessment/Évaluation:

on cutoffs. Secondary outcomes include presence of (% above cutoff) and severity (mean score) for postpartum depression, anxiety, stress, mean prenatal and postnatal self-efficacy, sense of mastery, self-esteem, sleep, relationship quality, coping, APGAR, birth weight, maternal-infant attachment, infant behavior, parenting stress and cost effectiveness.

The sample size for the RCT appears adequate, but the structural equation modeling appears insufficient. The rule of thumb as stated is not quite accurate: it is that one should have at least 10 cases per level of a variable, not per variable (a variable with 3 levels requires 20 participants). Further, the definition of case for a dichotomous outcome such as the secondary outcomes is the number of positive cases, not the total population. The explanation is simple: if you have 50 cases of maternal depression, and you have 10 variables with 3 categories (30 categories), that means you are going to have too many of the cells in the table with very few or 0 cases, and this makes the estimates unstable.

Analyses will be intention to treat but the sections dealing with missing data, compliance-based analyses and other complex issues require more explanation. The multiple regression models appear to be using a form of forward stepwise regression without attention to causal relations of variables or questions of total causal effect versus effect decomposition into direct and indirect effects. There are planned subgroup analyses for number of CBT sessions, anti-depressant use, symptom clusters, severity of DASS21, additional health services, participant characteristics, mental health history and gestational age. These represent a mix of baseline and post-randomization variables. It is not clear how these results would be interpreted, which is important given that some of the variables appear to be affected by the intervention itself. As such, they appear more related to the structural equation modeling described in the Mechanisms section and it is not clear why they are included in this section at all.

Phase 2 methods are briefly described as semi-structured interviews, audio-taped and transcribed. Section 5.3 describes coding and analysis in 8 lines.

Feasibility

The applicants say 60 pregnant women per week are seen of which they expect to have ~10 eligible and 4 to participate. The study requires a research coordinator to be onsite. This means the research coordinator is in the physicians’ office 3 ½-days per week to recruit 4 patients per week. The online questionnaires would facilitate data collection and analysis, but also includes sensitive information. The applicants say attrition rates for the online CBI program are half of group CBT.

**Review Type/Type dévaluation:**	Committee Member 2/Membre de comité 2
**Name of Applicant/Nom du chercheur:**	KINGSTON, Dawn Aileen
**Application No./Numéro de demande:**	287111
**Agency/Agence:**	CIHR/IRSC
**Competition/Concours:**	2012-09-17 Operating Grant/Subvention de fonctionnement
**Committee/Comité:**	Health Services Evaluation & Interventions Research 2/Recherche en interventions et en évaluation dans les services de santé 2
**Title/Titre:**	Integrated Maternal Psychosocial Assessment to Care Trial (IMPACT): Intervening Early to Improve Maternal and Child Health

Assessment/Évaluation:

Patients are clearly not blinded to the intervention but there is an attempt to blind them to the expected effects of the intervention on mental health (this would depend on what they know about CBT). The applicants say that patients will be told not to tell their physicians, in order that physicians remain blind and do not alter their treatment. It is not clear that this will be possible. If mothers are feeling depressed, the physician who notices this will ask them what they are doing, and the CBT program has to be part of their answer. I don't see a resolution to this and the applicants will have to be careful about over-interpreting the study results. The outcomes are all self-reports.

The actual CBT program is not yet online and only in paper form. Converting paper to online is not a simple task. It would seem likely that problems will arise and pilot testing will take a long time. This would result in delays and increased cost if some personnel are hired early in the project. It would seem that a research assistant is not really required until this phase is completed. A detailed description of the personnel required in each of the phases of the time line would be helpful, even if just a figure.

Originality

The study appears to be original in using technology for efficient data collection, and delivery of an intervention. An effective online intervention has the potential for much larger impact.

Applicant

The PI obtained her PhD in 2009 and completed post-doctoral training in 2011. Most of her funding is as a co-investigator with her supervisors, with one $50k grant as PI. She has been very productive in publishing in 2012 since the end of her post-doc. The PI is supported by a team with more experience in grants and publications, with expertise in the relevant areas.

Impact of Research

It is not entirely clear how much low to moderate stress affects clinical outcomes and in that respect, the impact is uncertain. The delivery using an online model of care has the potential to reach a very large audience, and may be useful for other areas of mental health research as well.

**Review Type/Type dévaluation:**	Committee Member 2/Membre de comité 2
**Name of Applicant/Nom du chercheur:**	KINGSTON, Dawn Aileen
**Application No./Numéro de demande:**	287111
**Agency/Agence:**	CIHR/IRSC
**Competition/Concours:**	2012-09-17 Operating Grant/Subvention de fonctionnement
**Committee/Comité:**	Health Services Evaluation & Interventions Research 2/Recherche en interventions et en évaluation dans les services de santé 2
**Title/Titre:**	Integrated Maternal Psychosocial Assessment to Care Trial (IMPACT): Intervening Early to Improve Maternal and Child Health

Assessment/Évaluation:

Budget

Most of the budget is related to salaries. As above, the efficiency of having a research assistant in a medical office 3 half days per week to recruit 4 patients per week is questionable. There is a budget for both a Masters and a PhD student. 4 tablets are requested to ensure women can complete the survey before their appointment. However, there are only 4 patients recruited per week. It would seem eligibility determination is very fast and it is only the full details that would require time. Two tablets would seem to suffice.

**Review Type/Type dévaluation:**	SO Notes /Notes de l'agent scientifique
**Name of Applicant/Nom du chercheur:**	KINGSTON, Dawn Aileen
**Application No./Numéro de demande:**	287111
**Agency/Agence:**	CIHR/IRSC
**Competition/Concours:**	2012-09-17 Operating Grant/Subvention de fonctionnement
**Committee/Comité:**	Health Services Evaluation & Interventions Research 2/Recherche en interventions et en évaluation dans les services de santé 2
**Title/Titre:**	Integrated Maternal Psychosocial Assessment to Care Trial (IMPACT): Intervening Early to Improve Maternal and Child Health

Assessment/Évaluation:

The proposal addresses an important clinical question. The internet-based intervention is novel. The proposal shows a clear progression from previous work. Pilot data is available indicating that the intervention is acceptable. The research question is clear and the use of a clinical trial with an integrated economic analysis is appropriate and is a strength. The range of tools is appropriate. There is good potential to generate new knowledge. The principal investigator and the team are very strong. If successful, the intervention can be widely integrated into primary care. The KT plan engages end users and decision makers and is very good.

The intervention is unblinded and the outcome is self-reported, but there is a good description of methods to address potential biases. The use of a screening tool as a method to diagnose a vulnerable population could be problematic.
